# Clinical features and risk factors of neonatal late-onset sepsis complicated by purulent meningitis

**DOI:** 10.3389/fped.2026.1704622

**Published:** 2026-01-22

**Authors:** Yuan Gu, Haifeng Geng, Huawei Wang, Xueping Zhu

**Affiliations:** 1 Department of Neonatology, Children’s Hospital of Soochow University, Suzhou, China; 2 Department of Pediatric, The First People’s Hospital of Taicang City, Suzhou, China

**Keywords:** clinical fetures, late-onset sepsis, neonate, purulent meningitis, risk factors

## Abstract

**Objective:**

To explore the clinical characteristics of neonatal late-onset sepsis (LOS) and analyze the independent risk factors for secondary neonatal purulent meningitis (NPM).

**Methods:**

This retrospective case-control study included infants diagnosed with LOS at the Children's Hospital of Soochow University between January 2018 and December 2023. The study divided the patients into two groups: the NPM group and the non-NPM group, based on the presence of secondary purulent meningitis. Clinical characteristics, laboratory markers, pathogen distribution, and treatment regimens were compared between the two groups. Independent risk factors were identified through multivariable logistic regression analysis, and a receiver operating characteristic (ROC) curve was used to evaluate the predictive performance.

**Results:**

A total of 453 LOS patients were included, with 98 (21.6%) cases in the NPM group. Compared to the non-NPM group, the NPM group exhibited a higher frequency of prolonged fever (>3 days), fever peak >39 °C, tachypnea, seizures, irritability, poor feeding, and bulging anterior fontanel (all *P* < 0.05). Laboratory tests showed elevated procalcitonin (PCT) in the NPM group, while albumin, cholinesterase, glycocholic acid, and creatine kinase (CK) levels were decreased (all *P* < 0.05). Blood culture results revealed that the NPM group had a significantly higher proportion of non-Group *B Streptococcus* and Enterobacter cloacae, but a lower proportion of *Staphylococcus aureus* (*P* < 0.05). Multivariable analysis identified prolonged fever (>3 days), fever peak >39 °C, tachypnea, PCT >10.50 ng/mL, and CK <200 U/L as independent risk factors for LOS complicated by NPM. ROC analysis showed that the combined prediction model had an AUC of 0.804 (95% CI: 0.751–0.856), with a sensitivity of 75.24% and specificity of 72.83%, which outperformed the individual predictors for predicting NPM.

**Conclusion:**

Prolonged high fever, abnormal respiration, elevated PCT, and decreased CK levels are important independent predictors of LOS complicated by NPM. The combined prediction model demonstrates high diagnostic efficacy, providing a useful reference for early identification of high-risk infants and the development of personalized intervention strategies.

## Introduction

Neonatal sepsis (NS) is a clinical syndrome of systemic illness in neonates, most commonly caused by bacterial bloodstream infection. It is one of the leading causes of neonatal death and disability (1, 2). Global data shows the incidence of neonatal sepsis is approximately 2,202 cases per 100,000 live births, while in China, the incidence ranges from 4.5‰ to 9.7‰, with a mortality rate of 11% to 19% (3–5). Based on the timing of onset, neonatal sepsis can be classified into early-onset sepsis (EOS) and late-onset sepsis (LOS). EOS occurs within the first three days after birth and is closely related to perinatal factors (6). With the improvement of maternal and child healthcare awareness, the incidence of EOS has decreased. In contrast, LOS is more common among infants with sepsis. LOS presents with atypical and diverse clinical features and lacks specificity (7, 8).

Neonatal Purulent Meningitis (NPM), also referred to as neonatal bacterial meningitis, is an acute purulent infection of the meninges caused by pyogenic bacteria (9). It is a frequent and severe complication of neonatal sepsis and remains an important contributor to mortality and long-term neurodevelopmental sequelae in early life (10). The incidence and mortality rates of NPM vary significantly across different regions. In developed countries such as those in Europe and North America, the incidence is approximately 0.3 per 1,000 live births, with a mortality rate of 10% to 15%. However, in developing countries, the situation is more severe, with incidence rates ranging from 0.8 to 6.1 per 1,000, and mortality rates can be as high as 40% to 58% (11–13). Therefore, focusing on the neonatal LOS population and identifying the clinical risk factors for potential progression to NPM at an early stage is critical for improving the prognosis of these infants. While some studies have concentrated on the microbiological characteristics and certain laboratory markers, there remains a lack of systematic analysis based on clinical data and stable predictive models (14, 15).

Therefore, this study aims to conduct an in-depth investigation into the clinical characteristics, laboratory parameters, and microbiological results of LOS patients to identify the risk factors for the development of secondary NPM. This will be crucial for early diagnosis, precise treatment, and improving prognosis. A retrospective case-control approach will be used to systematically analyze LOS patients treated at the Children's Hospital of Soochow University over six years. The goal is to explore the risk factors for LOS complicated by NPM and provide theoretical support for the early identification and improvement of prognosis in these infants.

## Methods

### Study subjects and grouping

This study is a retrospective case-control study that collected clinical data of neonatal sepsis (NS) patients hospitalized in the Department of Neonatology at the Children's Hospital of Soochow University from January 1, 2018, to December 31, 2023. The study included neonates who met the diagnostic criteria for late-onset sepsis (LOS) and had received treatment in accordance with the LOS clinical guidelines.

Exclusion criteria: (1) Cases in which the diagnosis was uncertain or could not be definitively confirmed based on available clinical manifestations and laboratory findings, or cases with suspected specimen contamination. (2) Hospital-acquired/healthcare-associated LOS. (3) Neonates diagnosed with genetic metabolic disorders and chromosomal diseases. (4) Received antibiotics prior to admission or whose clinical data were severely incomplete.

The subjects were divided into two groups: NPM group and non-NPM group, based on the presence of secondary purulent meningitis. Clinical characteristics and laboratory results of the two groups were compared.

This study was approved by the Medical Ethics Committee of the Children's Hospital of Soochow University (Ethical Approval No: 2023CS193). Written informed consent has been obtained from the patient's family, granting permission for the publication of their child's clinical data.

### Diagnostic criteria

The diagnostic criteria for neonatal sepsis (NS) were based on the guidelines for neonatal sepsis diagnosis formulated by the Neonatology Group of the Chinese Pediatric Society in 2019 (16). Confirmed diagnosis: compatible clinical manifestations plus a positive culture from blood or cerebrospinal fluid (CSF) (or other normally sterile body fluids). Clinical diagnosis: abnormal clinical manifestations, together with any one of the following: (1) ≥2 positive non-specific hematologic (non-pathogen-specific) laboratory findings; (2) CSF findings consistent with purulent meningitis; (3) detection of pathogen DNA in blood.

According to the timing of onset, NS is classified into early-onset sepsis (EOS) and late-onset sepsis (LOS). EOS refers to sepsis occurring within the first 72 h of life, while sepsis occurring after 72 h is classified as LOS (16).

The diagnostic criteria for neonatal purulent meningitis (NPM) follow the standards outlined in the 5th edition of *Practical Neonatology* (6): (1) General clinical manifestations of NPM, specific signs (e.g., altered consciousness, eye abnormalities, signs of increased intracranial pressure, seizures, sepsis phenotype). (2) CSF pleocytosis, defined in this study as CSF containing white blood cell (WBC) of ≥ 20 cells/uL, neutrophil predominance in CSF, and supportive biochemical abnormalities, including elevated CSF protein and decreased CSF glucose (and/or decreased CSF-to-blood glucose ratio). (3) Positive cerebrospinal fluid (CSF) culture, and/or the presence of bacteria observed on CSF smear or Gram staining. A clinical diagnosis of NPM was established when criteria (1) and (2) were fulfilled, whereas fulfillment of all three criteria was considered the gold standard for definitive diagnosis. In this study, patients meeting either the clinical diagnosis or confirmed diagnosis were classified into the NPM group; therefore, the non-NPM group included infants with sepsis without meeting NPM criteria.

“Underlying conditions and primary infectious foci” were defined as baseline conditions or the primary infection focus documented prior to LOS onset, including patent ductus arteriosus (PDA), pneumonia, urinary tract infection, and intestinal infection. “Comorbidities” were defined as concurrent complications or organ dysfunctions present at LOS onset or during the LOS episode, including liver dysfunction, kidney dysfunction, thrombocytopenia, electrolyte disturbances, multiple organ dysfunction syndrome (MODS), disseminated intravascular coagulation (DIC), respiratory failure, and necrotizing enterocolitis (NEC), the diagnostic criteria are also based on the 5th edition of *Practical Neonatology* (6).

### Clinical data collection

Clinical data were obtained by reviewing electronic medical records and included the following: demographic information such as the infant's gender, gestational age, birth weight, delivery method, Apgar score, duration of premature rupture of membranes, maternal fever during pregnancy, chorioamnionitis, amniotic fluid contamination, and gestational hypertension or diabetes; clinical manifestations including fever, peak fever, seizures, bulging anterior fontanel, pallor, poor feeding, irritability, tremors, tachycardia, tachypnea, apnea, lethargy, abdominal distension, and vomiting; underlying primary diseases and comorbidities such as pneumonia, patent ductus arteriosus (PDA), liver and kidney dysfunction, electrolyte imbalances, urinary tract infections, and intestinal infections; laboratory data including the first blood routine, biochemistry, blood culture, and cerebrospinal fluid (CSF) culture results at the onset of infection symptoms.

### Statistical analysis

Data analysis was performed using SPSS 26.0. First, normality and homogeneity of variance tests were conducted for all continuous variables. Normality was tested using the Shapiro–Wilk method, and homogeneity of variance was assessed using Levene's method. For normally distributed data, mean ± standard deviation was used to express the data, and independent two-sample *t*-tests were performed. Non-normally distributed data were presented as median and interquartile range (P25, P75) and analyzed using the Mann–Whitney *U*-test. Categorical data were presented as frequencies and percentages (%), and the Chi-square test was used for analysis. Additionally, multivariable logistic regression analysis was performed to identify the risk factors for NPM, and receiver operating characteristic (ROC) curves were constructed using GraphPad. A significance level of two-sided *P* < 0.05 was set for all tests.

## Results

### Clinical baseline characteristics of LOS infants

From January 1, 2018, to December 31, 2023, a total of 20,231 neonates were admitted to the Children's Hospital of Soochow University, of which 786 were diagnosed with neonatal sepsis (NS) (3.89%), and 574 were diagnosed with late-onset sepsis (LOS) (2.83%). Among the LOS patients, 343 were male and 231 were female, with a male-to-female ratio of 1.48:1. The average gestational age was (37.00 ± 4.00) weeks, and the average birth weight was (3,051.82 ± 830.00) g. Fifty infants were small for gestational age (SGA). A total of 230 infants were delivered via cesarean section, and 344 were delivered vaginally. The average age of onset for LOS was (14 ± 9) days, with a median onset age of 13 (range 8, 20) days.

**Figure 1:**
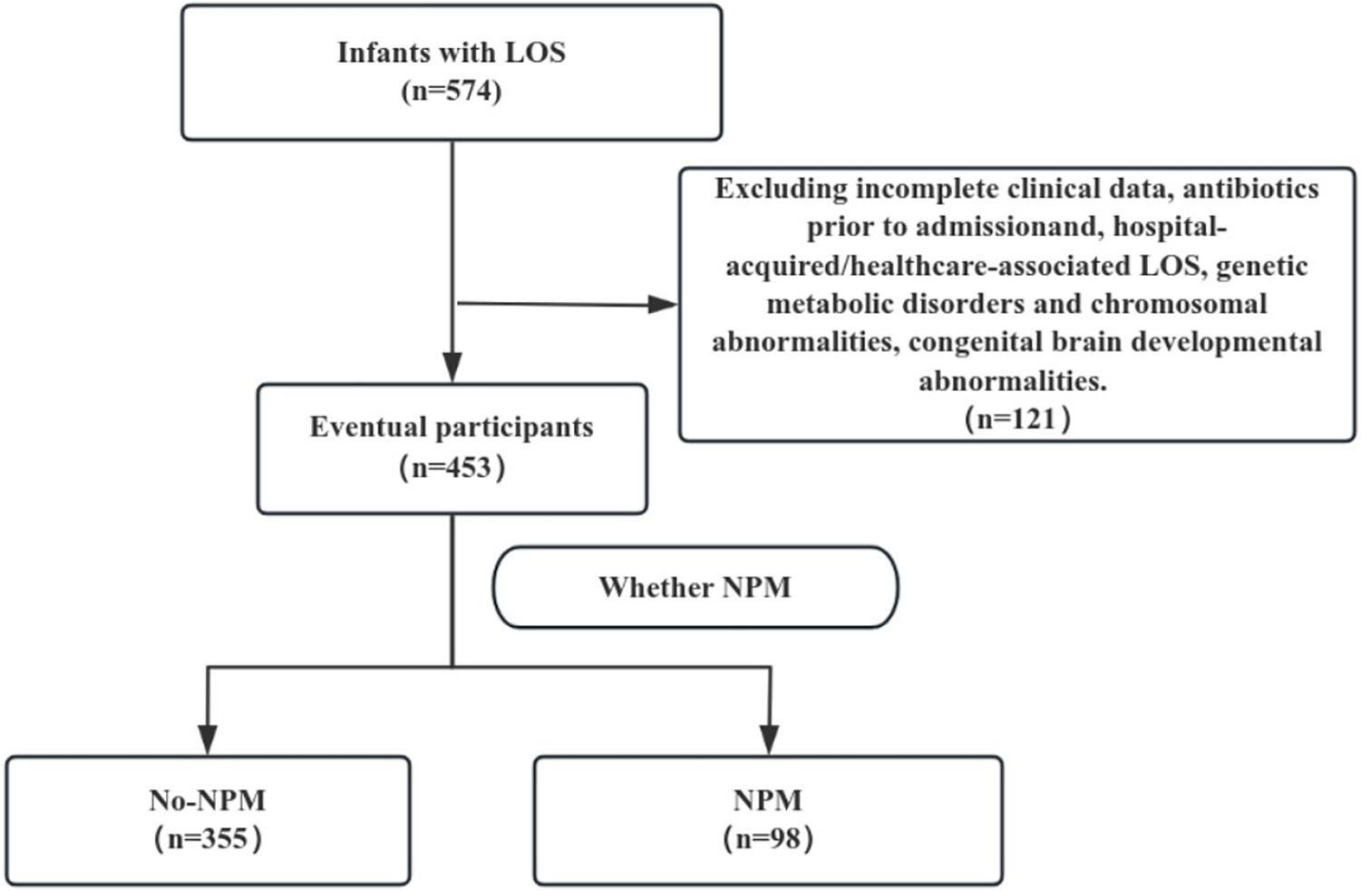
The study flowchart. LOS: late-onset sepsis; NPM: neonatal purulent meningitis.

A detailed study flowchart is shown in Figure 1. Of the 574 LOS infants, 111 cases with incomplete clinical data, received antibiotics prior to admissionand and hospital-acquired/healthcare-associated LOS, 7 cases with genetic metabolic disorders and chromosomal abnormalities, and 3 cases with confirmed congenital brain developmental abnormalities were excluded. Finally, 453 LOS patients met the inclusion criteria. Among these, 98 were diagnosed with secondary purulent meningitis (NPM group), and 355 were in the non-NPM group. The average age of onset for NPM was (16 ± 5) days, with a median onset age of 16 (range 9, 22) days. Among the 98 patients in the NPM group, cerebrospinal fluid cultures were positive in 23 cases (23.47%). The identified pathogens included *Escherichia coli* (*n* = 8), *Group B Streptococcus* (*n* = 6), *Enterobacter cloacae* (*n* = 3), *Enterococcus faecalis* (*n* = 2), *Staphylococcus* (*n* = 2), *Klebsiella pneumoniae* (*n* = 1), and *Listeria monocytogenes* (*n* = 1).

The clinical baseline data of the infants and mothers are detailed in Table 1. There were no significant differences between the two groups in terms of gender, gestational age, birth weight, delivery method, birth asphyxia, prolonged rupture of membranes ≥18 h, maternal antenatal fever, chorioamnionitis, amniotic fluid contamination, gestational hypertension, gestational diabetes mellitus, and antenatal antibiotics (*P* > 0.05).

**Table 1:** Comparison of clinical basic data between NPM group and Non-NPM group.

Characteristics		Non-NPM group (*n* = 355)	NPM group (*n* = 98)	*P*
Baseline characteristics of neonates				
Sex	Male	223 (62.82%)	58 (59.18%)	0.512
	Female	132 (37.18%)	40 (40.82%)	
Gestational age		38.02 ± 5.00	36.96 + 4.00	0.113
	<32w	11 (3.10%)	7 (7.14%)	0.071
	32w-36 + 6w	26 (7.32%)	10 (10.20%)	0.351
	≥37w	318 (89.58%)	81 (82.65%)	0.061
Birth weight		3,086.05 ± 823.22	3,003.11 ± 851.19	0.234
	<1,500 g	10 (2.82%)	6 (6.12%)	0.117
	1,500–2,499 g	29 (8.17%)	10 (10.20%)	0.930
	≥2,500 g	316 (89.01%)	82 (83.67%)	0.152
Mode of delivery	Cesarean section	141 (39.72%)	37 (37.76%)	0.725
	Vaginal delivery	214 (60.28%)	61 (62.24%)	
Asphyxia at birth		11 (3.10%)	4 (4.08%)	0.889
PROM≥18h		9 (2.54%)	5 (5.10%)	0.194
Maternal clinical characteristics during pregnancy				
	Maternal antenatal fever	6 (1.69%)	3 (3.06%)	0.657
	Chorioamnionitis	1 (0.28%)	0	1.000
	Amniotic fluid contamination	19 (5.35%)	8 (8.16%)	0.298
	Gestational hypertension	7 (1.97%)	3 (3.06%)	0.789
	Gestational diabetes mellitus	9 (2.54%)	3 (3.06%)	1.000
	Antenatal antibiotics	37 (10.42%)	12 (12.24%)	0.607

NPM neonatal purulent meningitis; PROM, prolonged rupture of membranes.

### Clinical characteristics, underlying primary diseases, and comorbidities

The clinical characteristics, underlying primary diseases, and comorbidities of the two groups are detailed in Table 2. When comparing the clinical features after admission, the NPM group had significantly higher proportions of fever lasting >3 days, peak fever >39 °C, poor feeding, lethargy, irritability, seizures, tachycardia, tachypnea, apnea, bulging anterior fontanel, and pathological jaundice compared to the non-NPM group (*P* < 0.05). However, no significant differences were observed between the two groups in terms of pallor, lethargy, abdominal bloating and vomiting (*P* > 0.05).

**Table 2:** Clinical characteristics, underlying primary diseases, and comorbidities in the NPM group and Non-NPM group.

Characteristics	Non-NPM group (*n* = 355)	NPM group (*n* = 98)	*P*
Clinical characteristics			
Fever lasting >3 days	15 (4.23%)	26 (26.53%)	0.000
Peak fever >39 °C	45 (12.68%)	26 (26.53%)	0.001
Pallor	28 (7.89%)	13 (13.27%)	0.100
Poor feeding	50 (14.08%)	22 (22.45%)	0.025
Vomiting	24 (6.76%)	17 (17.35%)	0.001
Irritability	16 (4.51%)	24 (24.49%)	0.000
Seizures	11 (3.10%)	9 (9.18%)	0.009
Tachycardia	13 (3.66%)	15 (15.31%)	0.000
Tachypnea	72 (20.28%)	43 (43.88%)	0.000
Apnea	16 (4.51%)	11 (11.22%)	0.013
Pathological jaundice	119 (33.52%)	46 (46.94%)	0.016
Bulging anterior fontanel	11 (3.10%)	15 (15.31%)	0.000
Lethargy	13 (3.66%)	7 (7.14%)	0.131
Abdominal bloating	21 (5.92%)	7 (7.14%)	0.590
Underlying conditions and primary infectious foci			
Patent ductus arteriosus	9 (2.54%)	6 (6.12%)	0.079
Pneumonia	69 (19.44%)	13 (13.27%)	0.903
Urinary tract infections	68 (19.15%)	10 (10.20%)	0.024
Intestinal infections	13 (3.66%)	2 (2.04%)	0.635
Comorbidities			
Liver dysfunction	18 (5.07%)	12 (12.24%)	0.011
Kidney dysfunction	69 (19.44%)	13 (13.27%)	0.903
Thrombocytopenia	13 (3.66%)	2 (2.04%)	0.635
Electrolyte disturbances	20 (5.63%)	5 (5.10%)	0.838
MODS	8 (2.25%)	2 (2.04%)	1.000
Shock	19 (5.35%)	3 (3.06%)	0.504
DIC	3 (0.85%)	0	1.000
Respiratory failure	26 (7.32%)	6 (6.12%)	0.681
Necrotizing enterocolitis	25 (7.04%)	6 (6.12%)	0.750

NPM, neonatal purulent meningitis; MODs, multiple organ dysfunction syndrome; DIC, disseminated intravascular coagulation.

When comparing underlying primary diseases and comorbidities between the two groups, the NPM group had a higher proportion of liver dysfunction compared to the non-NPM group, with a statistically significant difference (*P* < 0.05). The incidence of urinary tract infections was lower in the NPM group than in the non-NPM group (*P* < 0.05). There were no significant differences between the two groups in the incidence of PDA, kidney dysfunction, thrombocytopenia, electrolyte disturbances, intestinal infections, pneumonia, MODS, shock, DIC, respiratory failure, and NEC (*P* > 0.05).

### Laboratory tests and blood culture

The laboratory test results for both groups are detailed in Table 3. In the NPM group, the white blood cell (WBC) count <5 × 10^9^/L was significantly higher than that of the non-NPM group (*P* < 0.05). No significant differences were observed between the two groups in terms of WBC count, WBC count >20 × 10^9^/L, neutrophil ratio, lymphocyte ratio, hemoglobin, red blood cell distribution width, platelet count, platelet distribution width, and monocyte count (*P* > 0.05).

**Table 3:** Comparison of laboratory tests and blood culture pathogens between the NPM group and Non-NPM group.

Characteristics	Non-NPM group (*n* = 355)	NPM group (*n* = 98)	*P*
Laboratory tests			
WBC [×10^9^/L]	13.45 ± 8.12	12.48 ± 8.43	0.278
WBC>20 [×10^9^/L]	51 (14.37%)	10 (10.20%)	0.285
WBC<5 [×10^9^/L]	32 (9.01%)	19 (19.39%)	0.004
Neutrophil ratio [%]	54.80 ± 19.29	55.70 ± 19.04	0.669
lymphocyte ratio [%]	30.89 ± 18.91	29.32 ± 14.71	0.433
hemoglobin [g/L]	139.61 ± 31.31	139.93 ± 24.96	0.922
RDW [%]	15.32 ± 1.82	17.29 ± 20.18	0.317
Platelet count [×10^9^/L]	286.64 ± 134.61	278.34 ± 160.50	0.591
Platelet count<100 × 10^9^/L	28 (7.89%)	8 (8.16%)	0.929
Platelet count>300 × 10^9^/L	153 (43.10%)	32 (32.65%)	0.258
Platelet distribution width [%]	14.44 ± 2.66	14.50 ± 2.86	0.818
Monocyte count [×10^9^/L]	1.58 ± 1.42	1.96 ± 5,54	0.478
Total protein [g/L]	101.59 ± 33.56	104.51 ± 42.53	0.456
Albumin [g/L]	35.41 ± 3.91	34.34 ± 4.70	0.018
Prealbumin [g/L]	57.73 ± 17.32	57.58 ± 22.22	0.939
Aspartate aminotransferase [U/L]	56.72 ± 162.88	45.34 ± 53.42	0.479
Alanine aminotransferase [U/L]	23.40 ± 63.14	20.61 ± 22.17	0.655
Alkaline phosphatase [U/L]	225.87 ± 419.28	234.192 ± 432.13	0.857
Cholinesterase [U/L]	5,815.62 ± 1,814.43	5,277.78 ± 1,704.57	0.006
α-hydroxybutyrate dehydrogenase [U/L]	392.87 ± 361.18	363.17 ± 192.92	0.416
Lactate dehydrogenase [U/L]	522.89 ± 602.94	481.05 ± 271.28	0.487
Creatine kinase [U/L]	190.43 ± 275.28	125.28 ± 117.61	0.000
Creatine kinase isoenzyme [U/L]	17.91 ± 46.61	11.91 ± 27.66	0.258
Troponin T [ng/ml]	84.59 ± 205.57	41.64 ± 45.31	0.086
Glycocholic acid [mg/L]	14.75 ± 19.10	8.75 ± 7.60	0.001
C reactive protein [mg/L]	39.14 ± 48.84	47.46 ± 62.40	0.218
Procalcitonin [ng/ml]	9.55 ± 35.72	24.55 ± 35.34	0.001
Pathogens from blood culture			
*Escherichia coli*	25 (7.04%)	9 (9.18%)	0.476
*Group B Streptococcus*	5 (1.41%)	6 (6.12%)	0.007
*Staphylococcus*	38 (10.70%)	4 (4.08%)	0.045
*Listeria monocytogenes*	0	1 (1.02%)	0.490
*Klebsiella pneumoniae*	14 (3.94%)	2 (2.04%)	0.552
*Enterococcus faecalis*	5 (1.41%)	2 (2.04%)	1.000
*Enterobacter cloacae*	1 (0.28%)	4 (4.08%)	0.008
*Serratia marcescens*	1 (0.28%)	0	1.000
*Campylobacter jejuni*	1 (0.28%)	0	1.000
*Hemolytic Streptococcus*	1 (0.28%)	0	1.000
*Candida albicans*	2 (0.56%)	0	1.000

NPM, neonatal purulent meningitis; WBC, white blood cell.

When comparing blood biochemical markers, the NPM group had significantly higher procalcitonin (PCT) levels compared to the non-NPM group, with a statistically significant difference (*P* < 0.05). Additionally, albumin, cholinesterase, creatine kinase (CK), and glycocholic acid levels were significantly lower in the NPM group compared to the non-NPM group (*P* < 0.05). No significant differences were found between the two groups in terms of total protein, albumin, prealbumin, alanine aminotransferase (ALT), aspartate aminotransferase (AST), alkaline phosphatase, *α*-hydroxybutyrate dehydrogenase, lactate dehydrogenase, creatine kinase isoenzyme, troponin T, and C reactive protein (CRP) (*P* > 0.05).

Regarding the blood culture pathogen distribution, the NPM group showed a significantly higher proportion of Group *B Streptococcus* and *Enterobacter cloacae* compared to the non-NPM group, while the incidence of *Staphylococcus* was significantly lower in the NPM group (*P* < 0.05). No significant differences were observed between the two groups in the incidence of *Escherichia coli*, *Listeria monocytogenes*, *Klebsiella pneumoniae*, *Enterococcus faecalis*, *Serratia marcescens*, *Campylobacter jejuni*, *Streptococcus hemolyticus*, and *Candida albicans* infections (*P* > 0.05).

### Multivariable logistic regression analysis and ROC curve construction

Incorporating gestational age, birth weight, and other covariates that were clinically relevant and/or statistically significant in univariable analyses as independent variables, and the occurrence of secondary NPM as the dependent variable, we performed a multivariable binary logistic regression. The analysis revealed that the following factors were independent risk factors for LOS complicated by NPM: fever lasting >3 days (OR: 7.600, 95% CI: 2.416–23.910), peak fever >39 °C (OR: 3.164, 95% CI: 1.084–9.238), tachypnea (OR: 3.368, 95% CI: 1.412–7.123), high PCT levels (OR: 1.031, 95% CI: 1.014–1.049), and low CK levels (OR: 0.994, 95% CI: 0.990–0.999). These findings are detailed in Table 4.

**Table 4:** Multivariate logistic regression analysis of neonatal LOS and secondary NPM.

Characteristics	*P*	OR	95%CI
Procalcitonin	0.000	1.031	1.014–1.049
Creatine kinase	0.003	0.994	0.990–0.999
Tachypnea	0.004	3.368	1.412–7.123
Fever lasting >3 days	0.000	7.600	2.416–23.910
Peak fever >39 °C	0.035	3.164	1.084–9.238

NPM, neonatal purulent meningitis; LOS, late-onset sepsis.

**Figure 2:**
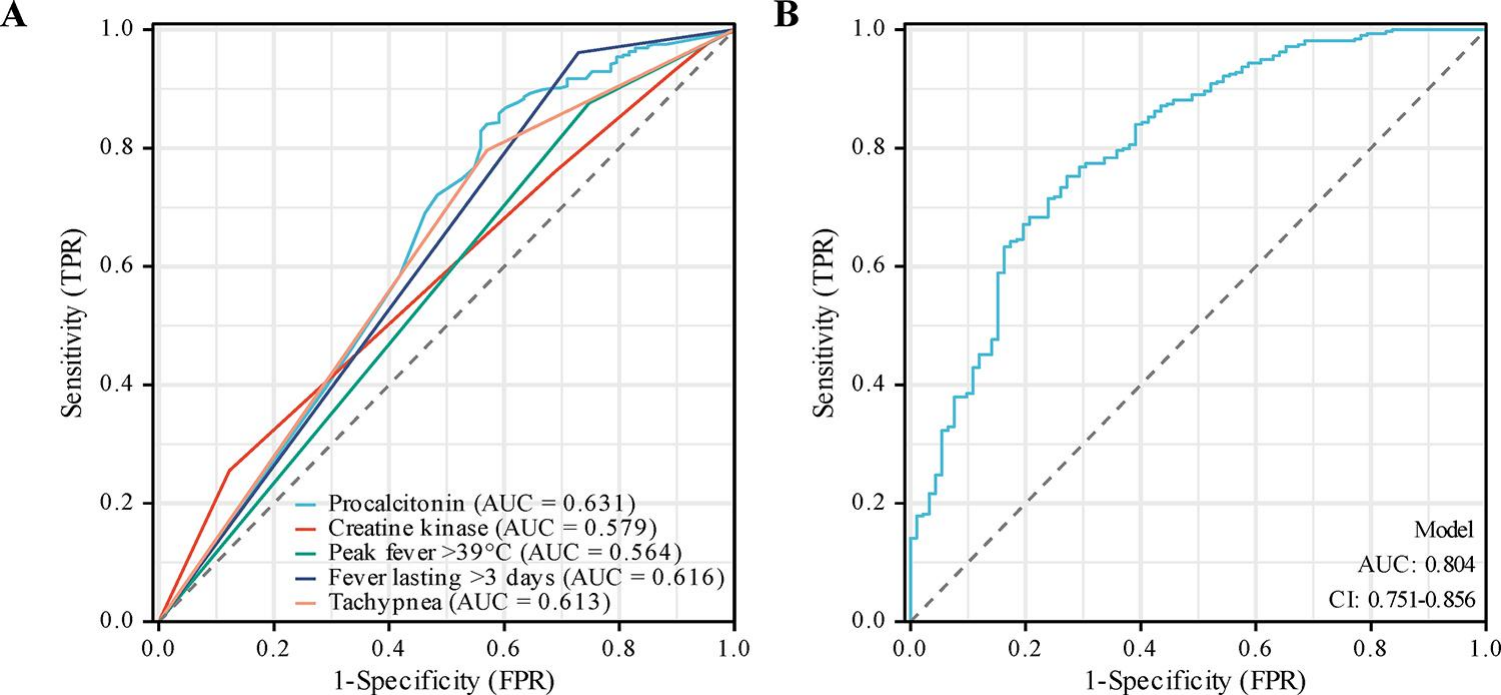
The ROC curve of predicting LOS in newborns with NPM based on independent risk factors. **(A)** Predictive ROC curves for NPM occurrence by clinical variables. **(B)** Joint predictive ROC curves for NPM occurrence. LOS, late-onset sepsis; NPM, neonatal purulent meningitis.

Using the control group as the reference level, ROC curves were plotted. The area under the curve (AUC) for PCT was 0.631 (95% CI: 0.563–0.700), with a cutoff value of 10.50 ng/mL. The AUC for CK was 0.579 (95% CI: 0.525–0.633), with a cutoff value of 200.00 U/L. The AUC for tachypnea was 0.616 (95% CI: 0.562–0.664), the AUC for fever lasting >3 days was 0.616 (0.573–0.660), and the AUC for peak fever >39 °C was 0.564 (95% CI: 0.520–0.609). The combined prediction model had an AUC of 0.804 (95% CI: 0.751–0.856), with a sensitivity of 75.24% and specificity of 72.83%, which was significantly higher than the individual predictions for each of the indicators. The details are shown in Table 5 and Figure 2.

**Table 5:** Parameters related to the prediction of secondary NPM in infants with LOS using logistic model.

Characteristics	AUC	*P*	95%CI	Cut-off	Sensitivity (%)	Specificity (%)
Procalcitonin	0.631	0.000	0.563–0.700	10.50	84.05	43.01
Creatine kinase	0.579	0.006	0.525–0.633	200.00	25.53	87.74
Tachypnea	0.613	0.048	0.562–0.664	——	——	——
Fever lasting >3 days	0.616	0.003	0.573–0.660	——	——	——
Peak fever >39 °C	0.564	0.005	0.520–0.609	——	——	——
Combined predictive	0.804	0.000	0.751–0.856	1.281	75.24	72.83

NPM, neonatal purulent meningitis; LOS, late-onset sepsis.

## Discussion

This study comprehensively collected clinical data and laboratory test results of the infants and explored the factors contributing to the occurrence of NPM in the context of LOS. The final analysis identified that fever lasting >3 days, peak fever >39 °C, tachypnea, PCT levels >10.50 ng/mL, and CK levels <200 U/L were risk factors for LOS complicated by NPM. Among these, fever lasting >3 days, peak fever >39 °C, tachypnea, and elevated PCT levels all indicated more severe conditions, which aligns with previous studies on neonatal sepsis. However, we are the first to report that relatively low levels of CK may indicate more severe illness. This may be related to the activation of an excessive inflammatory response or could be influenced by the timing of blood sample collection. Further follow-up to monitor the trend of these changes is needed.

NS typically manifests as fever, subnormal temperature, poor feeding, lethargy, reduced crying, tachypnea, jaundice, and abdominal distension. In severe cases, it can progress to lethargy, seizures, and even shock (16). In the case of LOS, the incidence of fever is higher, and a higher peak fever and longer duration often indicate a higher bacterial load and more severe inflammatory response in the body. This, in turn, increases the permeability of the blood-brain barrier, raising the likelihood of developing NPM (17, 18). In our cohort, the blood culture positivity rates were relatively low (26% in the NPM group and 28% in the non-NPM group). This finding is not unexpected in neonatal/infant sepsis, where culture-negative sepsis is common. Several factors may contribute to the limited yield of blood cultures, including prior antibiotic exposure before culture sampling, low inoculated blood volume, timing of sampling relative to intermittent/low-grade bacteremia, and the presence of localized infectious foci in which bloodstream invasion may be transient. Previous reports have shown that blood culture yield in neonatal sepsis can be modest (often ∼15%–20% in some settings) and is strongly influenced by blood volume collected, with improved sensitivity when adequate volumes are obtained (19–21). The high cesarean rate in this cohort likely reflects shared high-risk perinatal backgrounds. Its non-significant intergroup difference and absence as an independent NPM risk factor suggest cesarean delivery is more a clinical decision under high-risk conditions rather than a direct cause of NPM. These high-risk factors may jointly influence delivery mode and infection risk. Future prospective multicenter studies should adjust for perinatal confounders to clarify the independent role of delivery mode.

In this study, fever lasting more than 3 days and a peak fever above 39 °C were identified as independent risk factors for NPM in infants with LOS. Moreover, inflammatory markers in the NPM group were significantly higher than those in the general sepsis group. While there were no significant differences in white blood cell count between the two groups, the NPM group had a significantly higher proportion of infants with a decreased white blood cell count, suggesting that infants with LOS complicated by NPM experience more severe inflammation, which leads to bone marrow suppression. PCT has been established as a specific marker for severe bacterial infections and sepsis, closely correlating with the severity of bacterial infections (22, 23). In neonates, when PCT levels rise above the threshold of 0.5 ng/mL without an identifiable cause, sepsis or systemic inflammatory response syndrome (SIRS) should be considered (24). Furthermore, high levels of PCT have been proven to be closely associated with the occurrence and progression of NS (25). In this study, serum PCT levels in both groups were significantly higher than the normal threshold, and high serum PCT levels were found to be an independent risk factor for NPM in NS infants.

CK plays an essential role in cellular metabolism and energy homeostasis. Serum CK is found in skeletal muscle, heart, and brain tissues, and it exhibits significant biological activity (26). Elevated CK levels are closely associated with infectious diseases and often indicate the severity of organ and tissue damage (27, 28). Liu Xia et al. (29) found that serum CK levels were significantly increased in patients with viral meningitis, purulent meningitis, and tuberculous meningitis, with the most notable increase observed in VM. However, as research progressed and understanding of CK expanded, it was found that relatively low CK levels often suggest more severe disease and are closely associated with poor prognosis in certain conditions (30–32). In this study, serum CK levels were higher than normal in both the sepsis group and the NPM group. However, the NPM group showed lower serum CK levels, which were identified as an independent risk factor for the development of NPM in sepsis patients. This may be related to a decrease in CK activity caused by severe disruption of energy metabolism in the body (30).

This study is based on a single-center retrospective case analysis. Despite including a large sample size and spanning a long time period, the results may still be limited by regional differences, hospital treatment levels, and pathogen distribution, and the external validity needs further verification. Additionally, lumbar puncture was not routinely performed in all infants with late-onset sepsis, which may have led to underestimation of concomitant meningitis, particularly in cases without neurological manifestations. Furthermore, some missing case data may introduce selection bias, and laboratory markers show dynamic changes. This study only used single-time measurement results, which may not fully reflect the trend of these indicators over the course of the disease. Despite these limitations, the study systematically identified independent risk factors for NPM in LOS and developed a combined prediction model, which showed high predictive performance. Future multi-center, large-sample prospective studies are needed to further validate these findings and explore the potential value of dynamic monitoring and targeted intervention strategies for improving neonatal outcomes.

Based on a systematic analysis of the clinical characteristics of neonatal LOS, this study integrated traditional clinical symptoms (fever duration, peak temperature, tachypnea) with laboratory biochemical markers (PCT and CK) to construct a combined predictive model with high sensitivity and specificity for identifying high-risk populations for NPM secondary to LOS. Unlike previous studies that often relied on single inflammatory markers, this study found that low levels of CK independently predicted the occurrence of NPM, suggesting that energy metabolism may play crucial roles in disease progression. This finding offers a new perspective for exploring the pathological mechanisms of central infections associated with neonatal sepsis and provides a feasible tool for early screening, risk stratification, and precise intervention in clinical practice.

## Data Availability

The original contributions presented in the study are included in the article/Supplementary Material, further inquiries can be directed to the corresponding authors.
